# Angiogenesis-related gene NID2 profiling and immune infiltration in bladder cancer: prognostic implications and immunotherapy response

**DOI:** 10.3389/fimmu.2025.1615173

**Published:** 2025-08-29

**Authors:** Jinxia Cao, Wang Zheng

**Affiliations:** ^1^ Department of Hematology, Changde Hospital, Xiangya School of Medicine, Central South University (The First People’s Hospital of Changde City), Changde, Hunan, China; ^2^ Department of Organ Transplantation, Changde Hospital, Xiangya School of Medicine, Central South University (The First People’s Hospital of Changde City), Changde, Hunan, China

**Keywords:** angiogenesis, tumor microenvironment, TME, immunotherapy response, bladder cancer

## Abstract

**Background:**

Immunotherapy has progressively gained prominence as a cornerstone therapeutic modality across diverse oncological contexts, with its clinical efficacy intricately linked to the dynamic interactions between the tumor microenvironment (TME) and neoplastic cells. Central to this paradigm is angiogenesis—a quintessential hallmark of cancer—which not only sustains tumor growth but also orchestrates immunomodulatory networks within the TME, thereby profoundly influencing therapeutic responsiveness. However, in the field of bladder cancer (BC), the relationship among angiogenesis and prognosis, immunotherapy response, and immune cell infiltration remains to be further explored.

**Methods:**

To systematically uncover this relationship, we carried out an exhaustive assessment of 36 genes linked to angiogenesis (AAGs) and explored the relationship between angiogenesis and transcript, prognostic outcomes, as well as the infiltration of immune cells. By constructing an AAG_score, we quantified the angiogenic subtype characteristics of each patient. Subsequently, we evaluated the value of these characteristics in foreseeing BC prognosis and the response of treatment, and concurrently analyzed the performance of AAGs in diffuse large B-cell lymphoma for comparative study. Through RT-qPCR, CCK8 and other experiments, we verified the role of NID2 in bladder cancer.

**Results:**

This study explored different types of AAGs mutations in BC samples at the genetic level and elucidated the expression patterns of AAGs. Through in-depth analysis, we identified two distinct molecular subpopulations and found significant associations between AAG mutations and patients’ clinicopathological features, prognosis, and invasive TME. We found that patients with low AAG_score showed increased microsatellite instability, high mutation tendency, and immune motivation, and had a better prognosis. NID2 plays a role in promoting proliferation in bladder cancer and evaluate in diffuse large B-cell lymphoma. In addition, our study found a significant correlation between index of cancer stem cell and AAG_score in drug sensitivity.

**Conclusion:**

In summary, our study successfully identified prognosis-related AAG characteristics in BC patients. These characteristics not only contribute to a clearer understanding of TME properties but also provide an important basis for exploring more effective immunotherapy strategies.

## 1 Introduction

Tumor chemotherapy, as a therapeutic strategy that utilizes chemical drugs to suppress or eliminate cancer cells, occupies a pivotal position in the comprehensive treatment system of cancer (1, 2). Its core mechanism lies in the skillful use of drugs to precisely interfere with the DNA replication, protein synthesis, and metabolic processes of cancer cells, thereby effectively blocking their growth and division (3–5). TME is a multipart network interwoven by numerous cellular and molecular components, playing a decisive role in tumor initiation, response to treatment, and progression (6, 7). In the TME, the dense extracellular matrix and abnormal tumor blood vessels act as barriers, severely impeding the penetration of chemotherapeutic drugs into tumor tissues (8, 9). Meanwhile, in the TME, tumor-associated fibroblasts and immunosuppressive cells, including myeloid-derived suppressor cells (MDSCs), regulatory T cells (Tregs), and M2-type tumor-associated macrophages (TAMs), release signaling substances such as TGF-β and IL-6 that promote drug resistance (10, 11). These signaling molecules not only activate the anti-apoptotic pathways in cancer cells, enabling them to evade apoptosis, but also suppress the body’s anti-tumor immune response, impairing the immune system’s capacity to eradicate neoplastic cells (12). While new blood vessels provide the necessary nutrients for tumor growth, their leaky nature can lead to interstitial hypertension and promote extracellular matrix (ECM) remodeling, thereby creating a microenvironment conducive to tumor metastasis (13, 14). Previous studies have demonstrated that anti-angiogenic therapy can effectively inhibit tumor growth by suppressing tumor angiogenesis (15, 16). Given this, a comprehensive analysis of the intrinsic link between the TME and angiogenesis may help uncover diverse tumor immune phenotypes, thereby improving the ability to predict immunotherapy efficacy and providing a stronger theoretical foundation and practical guidance for precision cancer treatment.

Bladder cancer (BC), as one of the predominant malignant neoplasms in the urological system, also ranks among the ten most prevalent tumors in the body (17). In China, BC consistently has the highest incidence rate among genitourinary tumors (18). This condition can manifest at any stage of life, and its incidence is directly correlated with advancing age, peaking notably in the 50–70-year age group (19). Regarding gender disparities, the occurrence of BC is roughly three to four times more prevalent in males compared to females (20). Pathologically, BC comprises several types, including adenocarcinoma, urothelial carcinoma, and squamous cell carcinoma of the bladder (21). Notably, urothelial carcinoma is the most frequent, representing over 90% of all BC cases. The treatment approach for BC varies according to disease stage. For patients with muscle-invasive urothelial carcinoma, a standard treatment strategy involves neoadjuvant chemotherapy combined with surgery (22). For metastatic bladder cancer, chemotherapy is the primary treatment modality (23). Although there are currently numerous personalized chemotherapy regimens, the treatment outcomes remain unsatisfactory, with limited efficacy. Therefore, exploring new therapeutic targets has become a critical focus in the current field of BC treatment.

Once BC loses growth constraints, it will proliferate uncontrollably, and this process relies on nourishing blood vessels to deliver essential nutrients. The formation of new blood vessels significantly increases the vascular surface area available for tumor cells to invade (24). As a result, tumor cells are more likely to reach an effective number sufficient for metastasis through the bloodstream. Therefore, angiogenesis holds significant importance in the progression of tumor metastasis. In recent years, anti-angiogenic therapy has emerged as a prominent area of focus in anti-tumor treatment research (25, 26). This therapy focuses on inhibiting blood vessel formation in tumors, fundamentally weakening the tumor’s progression and spread capabilities by cutting off its nutrient supply and metastatic pathways, thereby achieving the goal of tumor treatment. In the practice of BC treatment, anti-angiogenic therapy has also demonstrated promising application prospects and has achieved certain research progress.

Currently, research on the association between angiogenesis-related genes and BC remains limited. With this in mind, we collected transcriptomic and single-cell data on angiogenesis-associated genes and BC, and performed an integrative analysis to explore disease progression, prognosis, TME characteristics, and treatment response in BC patients. In this study, we identified two distinct angiogenic subpopulations in BC. We therefore comprehensively evaluated the prognostic significance, molecular characteristics, and extent of immune cell infiltration in these two clusters. In addition, through a series of in-depth analyses, we developed a highly valuable AAG_score system capable of accurately predicting clinical outcomes and immunotherapy response in BC patients. We anticipate that our study will provide robust support for the development of effective immunotherapy strategies against BC.

## 2 Materials and methods

### 2.1 Data achievement

This study was conducted from the National Cancer Institute (NCI) and the National Human Genome Research The Institute (NHGRI) obtained Bulk transcriptome data for bladder cancer (BLCA cohort) and diffuse large B-cell lymphoma (DLBCL cohort). The BC single-cell transcriptome data (8 cases of tumors and 3 cases adjacent to cancer) were downloaded from PRJNA662018. Obtain CNV files, somatic mutation data, as well as the corresponding clinicopathological information from the BLCA and diffuse large B cell lymphoma(DLBC) project of National Cancer Institute (NCI). Angiogenesis-related gene set (AAGs) from the Molecular Signatures Database (MSigDB, https://www.gsea-msigdb.org/gsea/msigdb/cards/HALLMARK_ANGIOGENESIS) Database for a total of 36. The flowchart of this study was generated using Biorender.

### 2.2 Comprehensive characterization of AAGs in BC: expression, PPI network, and genomic alterations

Firstly, we used the limma package to conduct differential expression analysis of AAGs in cancer and adjacent tissues of the BLCA dataset. The screening criteria for differentially expressed AAGs were: log2 |Fold Change| >1.5 (log2 |FC| >1.5), P<0.05. And visualize the volcano map and box plot. To explore the interaction relationships of these genes, the protein-protein interaction network (PPI) of these genes was constructed using the STRING database. Subsequently, the single nucleotide mutations of these genes in the BLCA dataset were explored and a waterfall chart was created to visualize the mutation types and composition. We also used lollipop charts to present and explore the copy number variation (CNV) of AAGs in the BLCA dataset. Finally, we explored the expression of correlation in the BLCA cohort by AAGs and made a network graph for visualization.

### 2.3 AAGs-based consensus clustering analysis

Utilize algorithm of consensus clustering(k-means method) to identify unique AAGs profiles. Next, determination of both the optimal number(k values) of clusters and the consensus level was facilitated by “ConsensuClusterPlus” package. To ensure the robustness and stability of these clusters, perform 1,000 iterations of the clustering process (27). Subsequently, a consensus clustering matrix was drawn to visualize the clustering effect. To verify the grouping efficacy of the clustering results, we used principal component analysis (PCA) to observe the dispersion of the two clusters on the mathematical two-dimensional plane. Furthermore, Kaplan-Meier analysis, facilitated by the R packages “survminer” and “survival”, is employed to evaluate disparities in OS across distinct clustering patterns (28). We also analyzed the expression of AAGs and the age distribution among different clusters and generate heat map for visualizations. We also used GSVA to analyze the differences in biopathway enrichment among different clusters.

Then, we evaluated the differences in the abundance of 23 immune cell infiltrations among different clusters and visualized them with box plots. Subsequently, we compared the expression level differences of CD274, CTLA4 and PDCD1 among the clusters. We evaluated the differences in tumor microenvironment (TME) scores among clusters based on the ESTIMATE algorithm (29).

### 2.4 Unsupervised clustering based on prognostic AAGs

We calculated the differential genes between Cluster_1 and Cluster_2 based on the Limma R package and screened the prognosis-related genes using unicox. Then, Gene Ontology (GO) and Kyoto Encyclopedia of Genes and Genomes (KEGG) enrichment analyses were conducted based on these genes, and the pathway enrichment was visualized using bubble plots. Subsequently, based on the prognosis-related differences AAGs obtained through screening, unsupervised cluster analysis was conducted again to obtain gene_clusters. First, we explore the differences in overall survival (OS) among clusters. Subsequently, we analyzed the distribution differences of AGE, STAGE and gene expression levels among the clusters and visualized them with heat maps. Finally, we made a box plot to show the expression differences of AAGS among gene_clusters.

### 2.5 Establishment of the angiogenesis-associated gene score

Based on the prognostic differences AAGs obtained through 2.4 screening, we used the Least Absolute Shrinkage and Selection Operator (LASSO) +multiCox algorithm to establish a prognostic model for the BLCA dataset. The parameter is selected as lambda.min, and finally the AAGs score is formed. The formula of the relevant algorithm is as follows:


$$\text{AAG}\_\text{score}=\sum_{i=1}^{n}\left[Exp_{gene_{i}}*\beta_{i}\right]$$

Among them, $Exp_{gene_{i}}$ represents the expression level of the model gene, and *β_i_* represents the coefficient corresponding to the model gene. After the calculation model was established, we calculated the scores for each patient in the cohort. Based on the median score, we classified all patients into high-risk groups and low-risk groups. Subsequently, we drew a Sankey plot to observe the composition relationship among the AAG cluster, gene cluster and risk group, as well as their composition in the survival state. We also compared the differences in risk scores between different AAG clusters and gene clusters and represented them with box plots. Subsequently, we conducted a survival curve to demonstrate the OS differences between the two risk groups in the BLCA dataset. To verify the test efficacy of the model, we plotted receiver operating characteristic (ROC) curves for one year, three years and five years on the BLCA dataset, and calculated the area under each curve (AUC). We took an AUC greater than 0.6 to indicate that the model has good test efficacy. In addition, we have also drawn heat maps of the gene expression of patients under different risk scores and conditions.

### 2.6 Correlation analysis of AAG score and immunity

We analyzed the correlation between the risk score and the infiltration of 12 types of immune cells. Using the Pearson correlation coefficient method, we considered that there was a high correlation between the two when the absolute value of the correlation coefficient R was greater than 0.2. Subsequently, we calculated the estimate scores for the two risk groups, including stromal score, immune score and estimate score. Next, we will conduct a random forest analysis. We use a bar chart to show the relative importance of each feature in the prediction model. Select features with an importance greater than 0.4 for subsequent correlation analysis. We selected genes with an importance greater than 0.4 to analyze their correlation with the abundance of immune cell infiltration, using the Pearson correlation coefficient method and conducting heat map visualization. In addition, we also analyzed the expression differences of immune checkpoint related genes between the two risk groups.

### 2.7 Other comprehensive analyses of AAG score

First, we analyzed the differences in tumor mutational burden (TMB) between the two risk groups, and then analyzed the correlation between TMB and risk score and visualized it with a scatter plot. Next, we gave each patient a TMB score and divided them into a high TMB group and a low TMB group based on the median score. We conducted a survival curve analysis to examine the prognostic differences between the two groups. Subsequently, we alternately grouped the TMB and AAG score risk groups and compared the OS differences among these four groups. In addition, we separately analyzed the correlations between microsatellite instability (MSI), RNAss and AAG score, using the Pearson correlation coefficient method and conducting scatter plot visualization. Finally, we analyzed the somatic mutation characteristics of the high and low AAG_scores groups.

### 2.8 Analysis of drug sensitivity

First, we conducted an Immunophenoscore analysis between the two risk groups to assess their sensitivity to CTLA4 and PD1. Then we analyzed between the two groups. The sensitivity differences of the eight chemotherapy drugs were visualized by violin plots (30).

### 2.9 The processing and visualization of single-cell sequencing data

First, download the BC single-cell dataset for this paper from BioProject based on the project ID number: PRJNA662018. Cell Ranger 9.0.1 is obtained from the 10x Genomics official website, and the downloaded sra sequencing files are allied to the GRCh38 human reference genome. Quality control 1: Consistent with the quality control method in the original literature: Cells exhibiting UMI counts of less than 1000 or mitochondrial-derived UMI counts exceeding 10% are deemed to be of low quality and are consequently excluded. Additionally, to mitigate the potential presence of doublets, single cells in which more than 6000 genes are detected are also filtered out. Quality control 2: Remove RNA contamination using decontX, remove doublets using scrublet, and perform batch integration using harmony for multiple samples. We used UMAP to perform dimensionality reduction clustering on single-cell sequencing data and label their sources. Manual annotation is performed based on cell type-specific markers reported in previous literature. Gene set scoring: Based on the sc.tl.score_genes function in the Scanpy Python software (31). We present the expression of specific marker genes for each cell subpopulation through heat maps. Subsequently, we calculated the AAG score for each cell and visualized the score distribution through UMAP and bar charts.

### 2.10 Analysis of AAG score in non-solid tumors

In non-solid tumors, diffuse large B-cell lymphoma, we attempt to expand the application scope of AAG score. First, consistent consensus clustering is carried out based on AAG. The optimal number of clusters is selected based on delta area, cumulative distribution function (CDF) curve and proportion of ambiguous cluster, and heat map visualization is performed. Subsequently, a heat map was created to display the age distribution and gene expression among each cluster. We used PCA to verify the classification effect of the cluster classification method and calculated the survival curve to determine the survival differences among various clusters.

### 2.11 Cell culture and transfection

In this study, the human urothelial cell line SV-HUC-1 and the human bladder cancer cell lines SW-1710, ECV-304, T24, and BC-3C were selected for wet experimental verification. These cell lines are all derived from the Cell Bank of the Chinese Academy of Sciences. The culture conditions were strictly standardized: The cell lines SV-HUC-1, SW-1710 and ECV-304 were cultured in the RPMI-1640 medium. The cell lines T24 and BC-3C were cultured in Dulbecco’s Modified Eagle Medium (DMEM) medium. 10% fetal bovine serum (FBS, KeyGEN, China) and 1% mixture of penicillin and streptomycin (Procell, China) were added to all the culture media. The cell culture flasks are placed in a cell incubator at 37°C containing 5% CO2. To ensure that the cells are in the logarithmic growth phase, we change the culture medium every 2 to 3 days.

For the two cell lines of SW-1710 and BC-3C, we conducted transfection experiments. By using siRNA designed by a biological company (GIMA Corporation, China), we knocked down the expression of NID2 in different cell lines and used negative control (NC) as a control. First, we used trypsin (KeyGEN, China) to isolate the cells from the culture flask and suspend them in the culture medium. Subsequently, the cells were counted and the concentration was calculated. They were uniformly inoculated into 6-well plates at a concentration of 3×104 per well, and the culture medium was supplemented to 2ml in each well. After the cells have adhered, mix siRNA and LipofectamineTM 3000 (Invitrogen, USA) in a certain proportion, let it stand at room temperature for 15 minutes, and then use a pipette to evenly add the mixture to the corresponding Wells. The medium was replaced 6 hours after transfection, and the subsequent experiment was conducted 48 hours after transfection.

### 2.12 RT-qPCR analysis

We detected the mRNA expression differences of NID2 among human urothelial cell line SV-HUC-1, human bladder cancer cell lines SW-1710, ECV-304, T24 and BC-3C by RT-qPCR. Meanwhile, the mRNA expression differences between the NID2 knockdown group and the NC group in the SW-1710 and BC-3C cell lines were compared to verify the transfection efficiency. The specific experimental steps are as follows: Firstly, the cells in the 6-well plate were digested using trypsin (KeyGEN, China), and the cell precipitate was collected after PBS washing and low-temperature centrifugation. Subsequently, follow the instructions and add 800μl of Trizol (Takara, Japan) to the precipitate for thorough cell lysis. After 5 minutes of ice bath, add 200μl of chloroform (SINOPHARM, China), equal volume of isopropyl alcohol (SINOPHARM, China), and anhydrous ethanol (SINOPHARM, China) in sequence. After each addition of an organic solvent, it is necessary to let it stand at low temperature, centrifuge and remove the upper layer of organic solution. After drying in a laminar flow hood for 50 minutes, feather-like RNA precipitates were obtained. The RNA precipitate was dissolved in 20μl of DEPC-treated water, and the RNA concentration was determined using Nanodrop2000 (purchased from Thermo, USA). Next, referring to the instructions, the PrimeScript RT kit (TaKaRa, Japan) was used to remove genomic DNA from RNA first, and then reverse transcription reaction was carried out to obtain the cDNA solution. Pre-mix the cDNA sample with the reaction reagent using the SYBR GreenER Supermix (TaKaRa, Japan) kit to ensure that each tube of the reaction system contains 18μl of reagent and 2μl of cDNA. Real-Time fluorescence quantitative PCR was performed on the 7500 Real-time PCR System (Thermo Fisher Scientific, USA). Taking GAPDH as the internal reference gene, a two-step amplification procedure was adopted: pre-denaturation at 95°C for 10 minutes; Subsequently, 45 cycles of denaturation at 95°C for 5 seconds and annealing at 60°C for 30 seconds were carried out. Each group of samples contains three duplicate Wells. Based on the normalized relative expression of β-actin, the relative expression of NID2 was calculated using the 2^-ΔΔct^ method.

### 2.13 CCK8 assay

We selected two cell lines, SW-1710 and BC-3C, and detected the proliferation ability of the cells by CCK-8 assay. Forty-eight hours after transfection, the cells were seeded into 96-well plates at a density of 6,000 per well and then returned to the incubator for cell adhesion. Three replicate Wells were set up for each group of experiments. After discarding the original culture medium, mix the CCK8 reagent (KeyGEN, China) with the complete culture medium at room temperature as per the instructions, and quickly add it to the 96-well plate with a pipette to ensure that the final volume of each well is 200μl. Completely wrap the well plate with aluminum foil to avoid light, incubate for 2 hours, and then measure the absorbance of each well at a wavelength of 450nm using an enzyme-labeled instrument. Repeat the above detection steps at the time points of 24, 48, 72 and 96 hours respectively.

### 2.14 Flow cytometry

Forty-eight hours after transfection, in the SW-1710 and BC-3C cell lines, we used flow cytometry to verify the effect of NID2 on apoptosis. Firstly, the cells were digested with trypsin (Beyotime, China) free of Ethylene Diamine Tetraacetic Acid (EDTA, Beyotime, China), and the cell precipitate was obtained by centrifugation at 2,000 rpm. Wash the cells three times with PBS and then transfer them to a flow tube. Based on the recommendations of the instruction manual, an appropriate amount of Fluorescein Isothiocyanate (FITC, Sigma-Aldrich, USA) and propidium iodide (PI, Biosharp, China) were added to each group. After staining in a 37°C water bath in the dark for 15 minutes, the cells were aspirated by flow cytometry. Repeat the above operations for each group three times.

### 2.15 Wound healing assay

After transfection treatment of the SW-1710 and BC-3C cell lines for 48 hours, a vertical line was slowly and uniformly drawn in the well using a 200μl pipette tip with the assistance of a ruler. Replace the tip before each scratch. After scratching, discard the culture medium and gently wash three times with PBS to discard the floating cells. Add 2ml of the basic medium without FBS to each well. Subsequently, the scratch was photographed with a microscope and the wound area was recorded when it was defined as 0 at this time. After 24 hours of culture in the cell incubator, take photos again and record the area of the healed wound. Calculate the percentage of scratch healing.

### 2.16 Statistical analysis

All the analyses in this article were conducted based on R software (V4.3.1). Unless otherwise specified, the R packages used for plotting are all plotted with ggplot2. The t-test was used for the test between the two groups. One-way analysis of variance was used for tests among multiple groups. The statistical significance P value is defined as P less than 0.05.

## 3 Results

### 3.1 Landscape of genetic mutations of AAGs in BC

**Figure 1:**
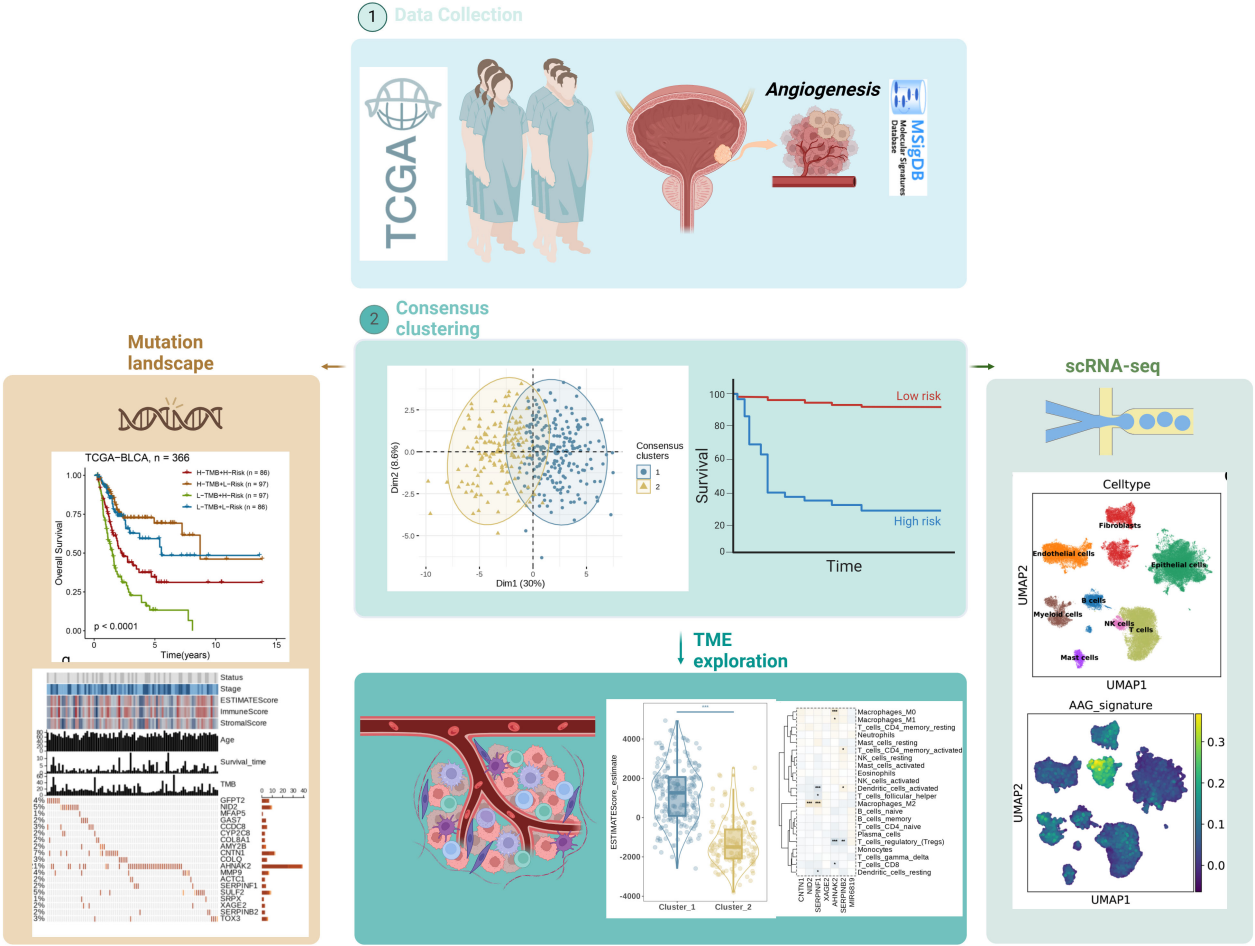
Study workflow diagram.

**Figure 2:**
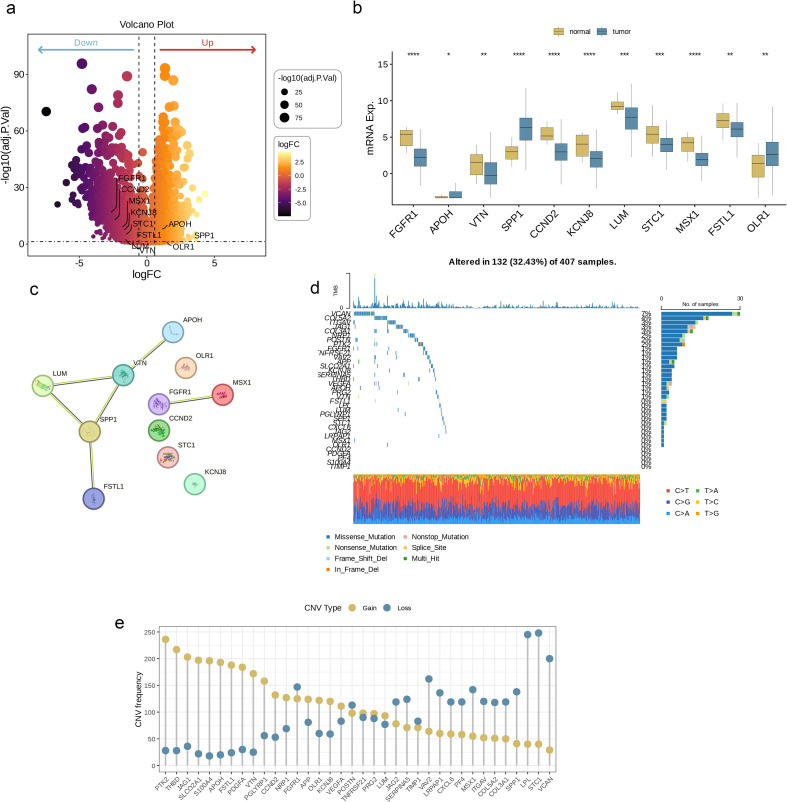
Genetic mutational landscape of AAGs in BC. **(a)** Volcano plot of differentially expressed genes (DEGs) between BLCA and normal tissues. **(b)** Expression distributions of differentially expressed angiogenesis-associated genes (DE-AAGs) between BLCA and normal tissues. **(c)** The PPI network acquired from the STRING database among the DE-AAGs. **(d)** Genetic alteration on a query of AAGs. **(e)** Frequencies of CNV gain, loss, and non-CNV among AAGs. (p< 0.05 *; p< 0.01 **; p< 0.001 ***; p< 0.0001 ****).

**Figure 1** illustrates the workflow of this study. By analyzing BLCA transcriptome data and combining it with AAGs collected from the Molecular Signatures Database (MSigDB) (**Figure 2a**), we successfully screened out 11 differentially expressed angiogenesis-associated genes (DE-AAGs) that regulate angiogenesis in BC (**Figure 2b**, p< 0.05). For the purpose of gaining a more profound information of the interrelationships among these genes, we developed a PPI network, which exhibited that there were relatively close connections among five of these genes (APOH、VTN、LUM、SPP1、FSTL1) (**Figure 2c**). Next, we investigated the copy number variations (CNVs) and the frequency of somatic mutations in AAGs within BC. Among 407 BC samples, 132 (accounting for 32.43%) had gene mutations (**Figure 2d**). Among the 36 AAGs, the VCAN gene had the highest mutation incidence, after that were the COL5A2 and ITGAV. Moreover, we also investigated the incidence of CNV mutations, which indicated that the 36 AAGs showed significant CNV alterations (**Figure 2e**). This study provides compelling evidence of significant alterations in the genomic landscape and the AAGs expression levels when comparing BC specimens to normal controls. This discovery indicates that AAGs could potentially play a crucial role in the initiation and progression of BC.

### 3.2 Generation of angiogenesis subgroups in BC

**Figure 3:**
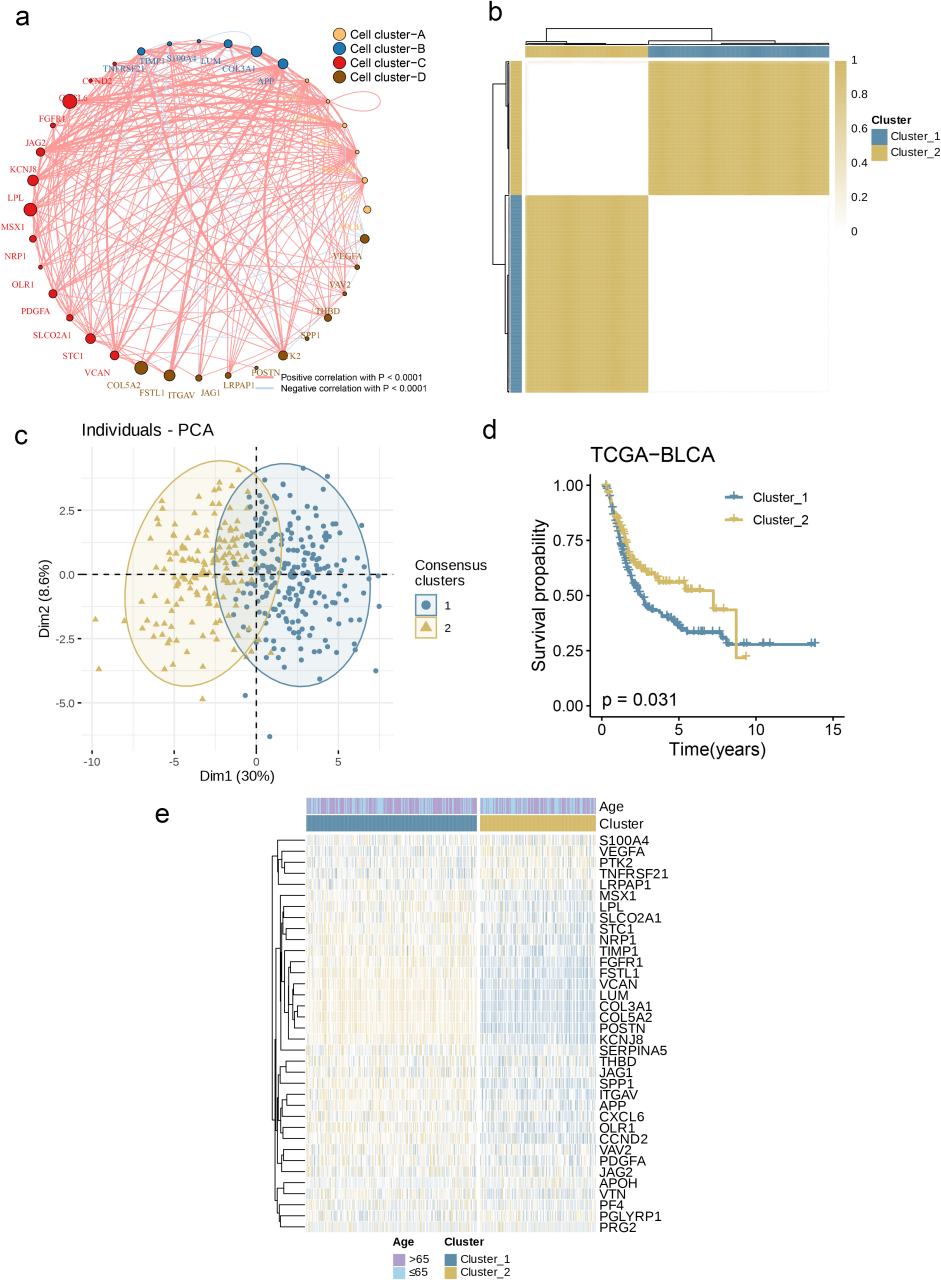
Generation of Angiogenesis Subgroups in BC. **(a)** A network of correlations including AAGs in the NCI cohort. **(b)** Consensus matrix heatmap defining two clusters (k = 2) and their correlation area. **(c)** PCA analysis indicating an obvious difference in transcriptomes between the two subgroups. **(d)** KM survival analysis showing 36 AAGs correlated with OS. **(e)** Differences in clinicopathologic characteristics and expression levels of AAGs between the two distinct subgroups.

Subsequently, we constructed a correlation network (**Figure 3a**) to present the interactions of AAGs in BC patients, the relationships among regulatory factors, and the survival significance of these relationships. To explore the underlying connections between AAGs and BC, we conducted an analysis focused on their expression levels, aiming to classify BC patients. Analysis showed that when the optimal number of clustering variables was set to 2 (**Figure 3b**), the BC patients in the research were clearly distributed into two subsections. The results of principal component analysis (PCA) further confirmed that there were good inter-group distribution characteristics between these two subgroups (**Figure 3c**). Furthermore, we conducted a comparison of the OS durations between patients in the two clusters and noted substantial disparities in survival outcomes——The prognosis of Cluster_1 is significantly worse than that of Cluster_2 (**Figure 3d**, p = 0.031). Additionally, as depicted in **Figure 3e**, the comparison of genomic expression patterns and clinical traits between the two clusters uncovered notable distinctions in AAGs expression and clinical characteristics.

### 3.3 Features of the TME across various subgroups

**Figure 4:**
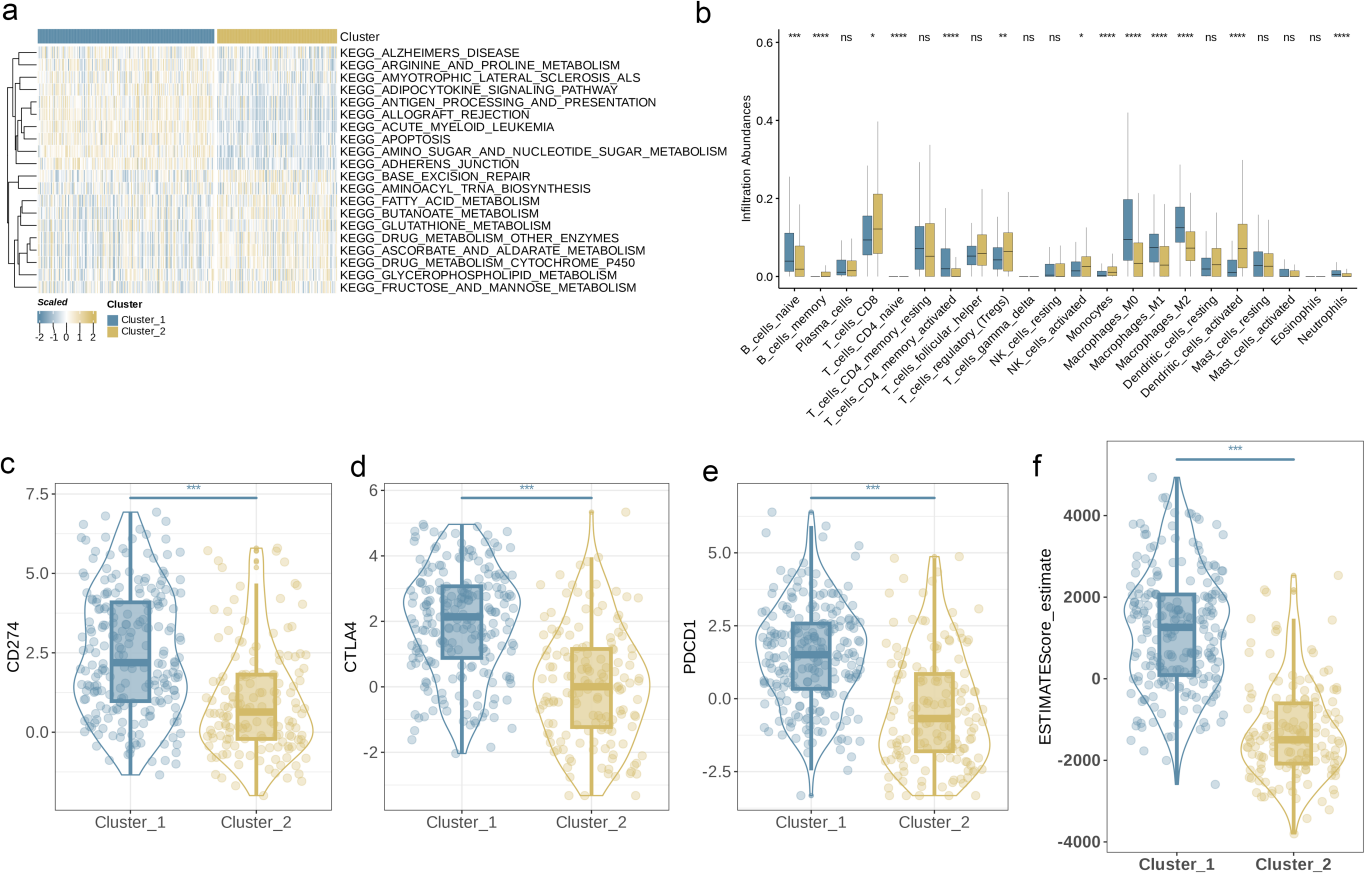
Characteristics of TME in Different Subgroups. **(a)** GSVA of biological pathways between two distinct subgroups. **(b)** Abundance of 23 infiltrating immune cell types in the two BLCA subgroups. **(c–e)** Expression levels of CD274, CTLA4, and PDCD1 in the two BLCA subgroups. **(f)** Correlations between the two BLCA subgroups and TME score. (p< 0.05 *; p< 0.01 **; p< 0.001 ***; p< 0.0001 ****; ns, no significance).

Subsequently, we used GSVA and found that Cluster 1 showed high activity in energy metabolism (such as arginine and proline metabolism) as well as antigen presentation and apoptosis; while Cluster 2 was more augmented in lipid metabolism and tricarboxylic acid cycle-related pathways (**Figure 4a**). To elucidate the association between AAGs and BC’s TME, we used the CIBERSORT algorithm to investigate the infiltration levels of 23 immune cell subsets in the two clusters. **Figure 4b** demonstrates significant variations in the enrichment of most immune cell types between the two clusters. Specifically, Cluster 1 exhibited significantly higher infiltration levels of CD4+ memory T cells, naive B cells, M0-type, M1-type, and M2-type macrophages, as well as neutrophils, compared to Cluster 2. Conversely, Cluster 2 was predominantly characterized by higher levels of memory B cells, CD8+ T cells, regulatory T cells, activated NK cells, monocytes, and activated dendritic cells (p< 0.05). Furthermore, we observed that the expression levels of three key ICPs, PD-1, PD-L1, and CTLA-4, were significantly higher in Cluster 1 relative to Cluster 2 (**Figures 4c–e**, p< 0.001). In this study, the tumor microenvironment score was utilized to assess the abundance of immune and stromal components within the TME. In addition, we calculated the tumor microenvironment scores, including stromal, immune, and estimate scores, for each cluster. The findings revealed that patients in Cluster 1 exhibited higher scores across these tumor microenvironment parameters (**Figure 4f**, p< 0.001).

### 3.4 Prognosis based on DEG-based gene subgroup identification

To delineate the mechanistic involvement of angiogenesis-associated genes (AAGs) in BC tumorigenesis and malignant progression, differential expression profiling was conducted using the limma computational framework, specifically quantifying inter-subgroup heterogeneity within angiogenesis-related molecular clusters.

**Figure 5:**
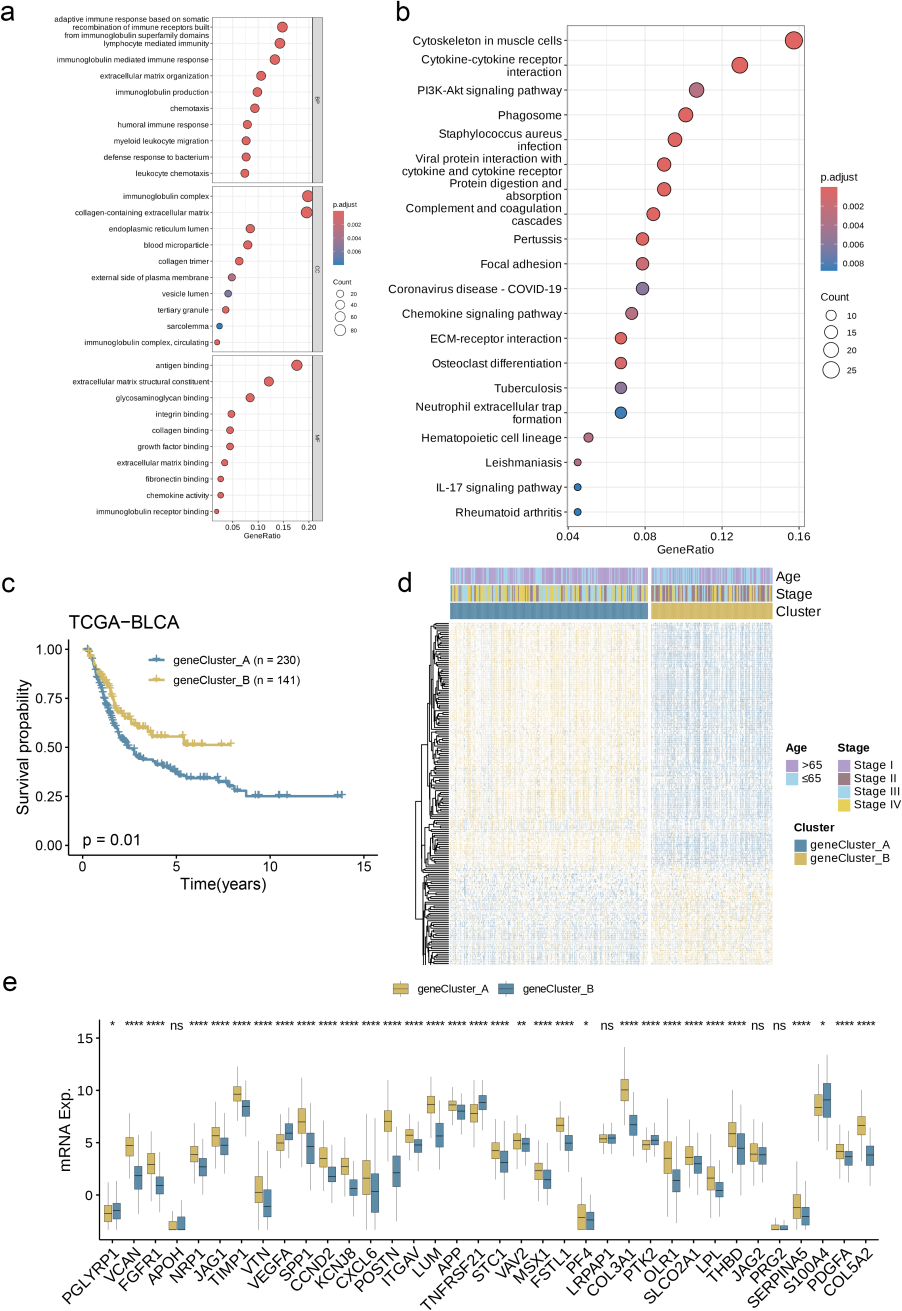
Prognosis Based on DEG-Based Gene Subgroup Identification. **(a, b)** GO and KEGG enrichment analyses of DEGs among two angiogenesis subgroups. **(c)** Kaplan–Meier curves for OS of the two gene clusters. **(d)** Relationships between clinicopathologic features and the two gene clusters. **(e)** Differences in the expression of 36 AAGs among the two gene clusters. (p< 0.05 *; p< 0.01 **; p< 0.001 ****; ns, no significance).

Through this analysis, we successfully screened out 234 AAG_DEGs related to the angiogenesis cluster and then conducted functional enrichment analysis on these genes. The analysis results showed that these DEGs related to the angiogenesis subgroup were primarily enhanced in biological processes closely related to cell adhesion, migration, proliferation, etc. (**Figure 5a**). Further KEGG analysis showed that there were a large number of signaling pathways that played important roles in maintaining homeostasis, defending against pathogen infection, regulating immune responses, and tissue repair (**Figure 5b**). Under specific conditions, abnormalities in these pathways and biological behaviors may trigger the occurrence and development of diseases, which fully proves that angiogenesis plays a key function in regulating tumor metastasis and affecting the progression of BC. In order to investigate the specific regulatory mechanisms in greater detail, patients were classified into two distinct clusters using an unsupervised clustering approach based on prognostic genes. Kaplan-Meier survival analysis indicated that Group A patients had the poorest overall survival (OS), while Group B patients exhibited significantly better OS outcomes (**Figure 5c**, p = 0.01). Additionally, we observed a significant association between angiogenesis gene cluster A and the advanced stages (stages III and IV) of BC (**Figure 5d**). Concurrently, the angiogenesis gene clusters exhibited notable differences in the expression of AAGs, aligning with our initial expectations for the angiogenesis subgroups (**Figure 5e**, p< 0.05).

### 3.5 Creation and verification of the prognostic AAGs_score

**Figure 6:**
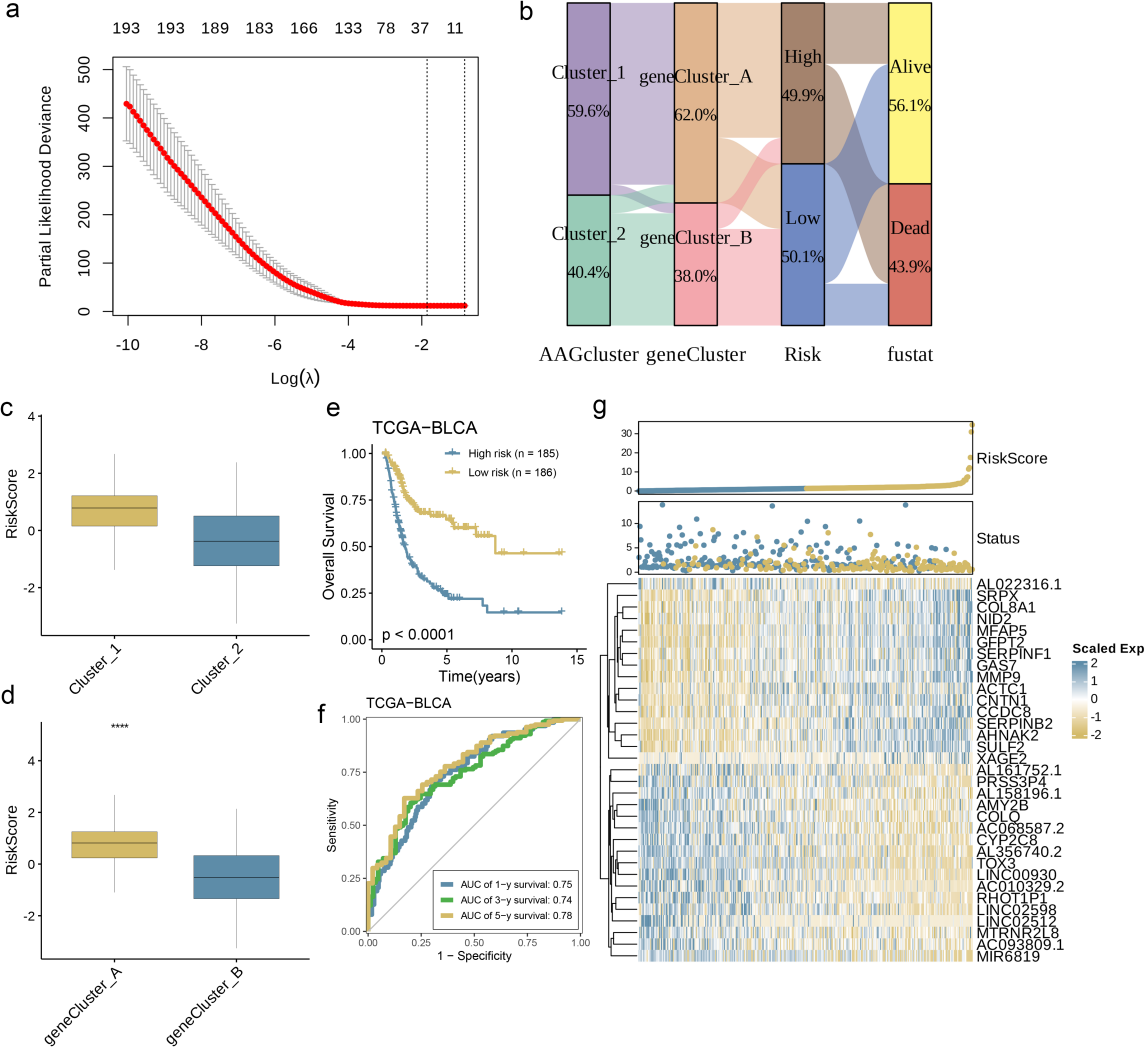
Development and Validation of the Prognostic AAG_Score. **(a)** The LASSO regression analysis and partial likelihood deviance on the prognostic genes. **(b)** Alluvial diagram of subgroup distributions in groups with different AAG_scores and clinical outcomes. **(c)** Differences in AAG_score between the two angiogenesis clusters. **(d)** Differences in AAG_score between the two gene clusters. **(e)** Kaplan–Meier analysis of the OS between the two groups. **(f)** ROC curves to predict the sensitivity and specificity of 1-, 3-, and 5-year survival according to the AAG_score. **(g)** Ranked dot and scatter plots showing the AAG_score distribution and patient survival status. Expression patterns of selected prognostic genes in high- and low-risk groups (p< 0.0001 ****).

The AAG_score was derived from DEGs that were linked to particular clusters. Initially, the univariate Cox (unicox) algorithm was employed to identify genes linked to prognosis. Subsequently, LASSO regression and multivariate Cox (multiCox) analysis were utilized to construct an optimal prediction model based on these genes (**Figure 6a**). Using this prediction model, we assigned scores to each sample within the BLCA dataset. As shown in **Figure 6b**, the distribution of patients is represented across two angiogenesis clusters, two gene clusters, and two distinct AAG_score groups. Further analysis revealed significant differences in the AAG_score between the angiogenesis clusters and gene clusters (**Figures 6c, d**, p< 0.0001). In particular, gene cluster A exhibited a higher AAG_score than gene cluster B, indicating that a reduced AAG_score could be linked to traits associated with immune activation. Based on the survival analysis results, we observed that a higher risk score was associated with poorer survival rates, regardless of whether the classification was based on angiogenesis clusters or gene clusters. Kaplan-Meier survival analysis of the training cohort further revealed that patients classified as low risk experienced significantly improved overall survival (OS) compared to their high-risk counterparts. (**Figure 6e**, p< 0.0001). Simultaneously, the area under the curve (AUC) for 1-year, 3-year, and 5-year OS was 0.75, 0.74, and 0.78, respectively (**Figure 6f**), indicating the model’s robust predictive performance. The risk map based on the AAG_score distinctly illustrated a negative correlation, with an increase in AAG_score associated with reduced overall survival (OS) and elevated mortality rates. Meanwhile, the expression heatmap of model-related genes revealed a close correlation between gene expression levels and prognosis time (**Figure 6g**). In summary, these results strongly suggest that the risk score of this model is negatively correlated with prognosis time and can accurately predict the prognosis of BC patients.

### 3.6 Evaluation of TME and checkpoints in different groups

**Figure 7:**
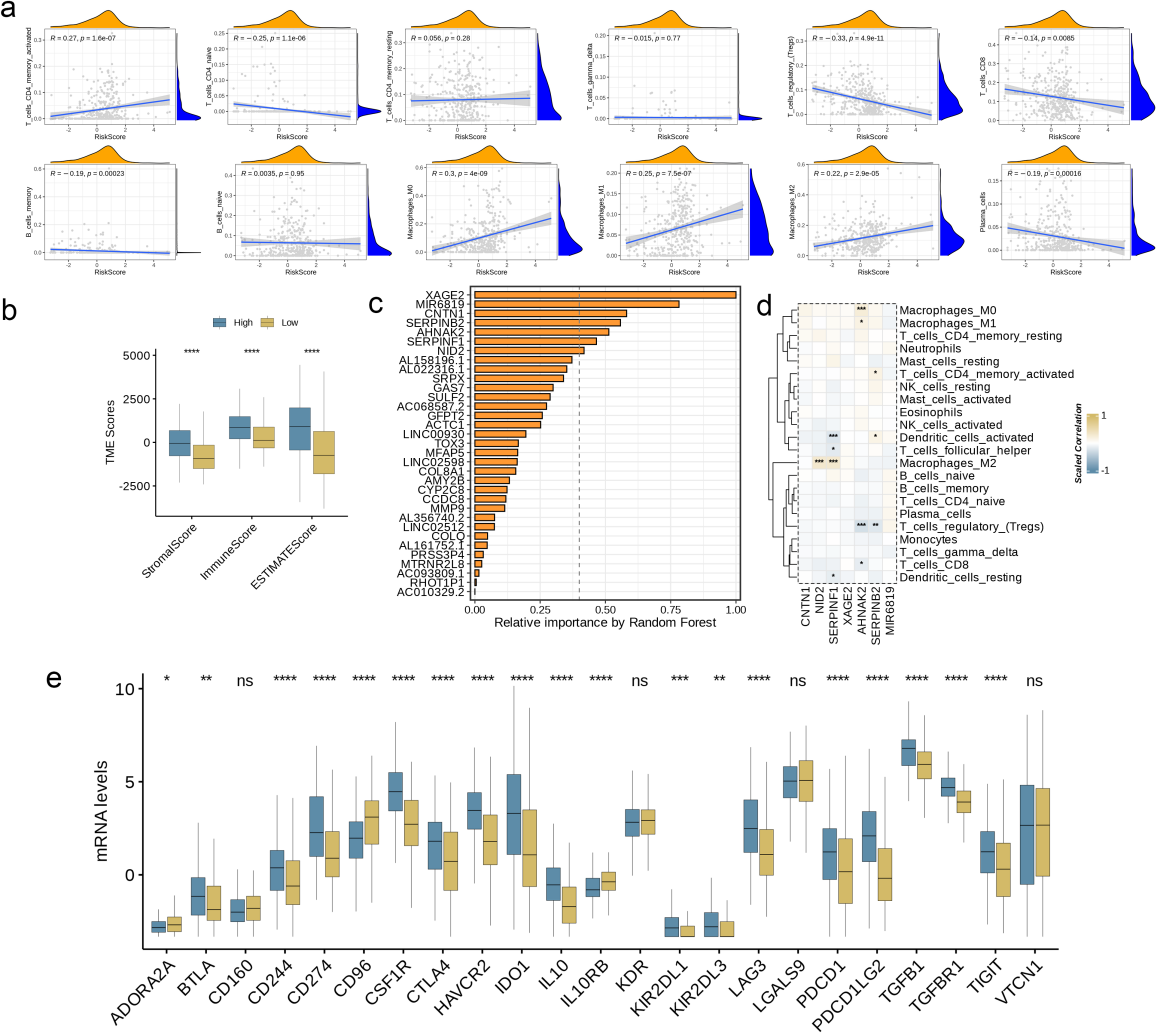
Evaluation of TME and Checkpoints in Different Groups. **(a)** Correlations between AAG_score and immune cell types. **(b)** Correlations between AAG_score and both immune and stromal scores. **(c)** Random forest analysis showing the relative importance of each feature in the prognostic model. Features with importance over 0.4 were selected for subsequent correlation analysis. **(d)** Correlations between the abundance of immune cells and selected genes in the prognostic model. **(e)** Expression of immune checkpoints in the high and low-risk groups. (p< 0.05 *; p< 0.01 **; p< 0.001 ***; p< 0.0001 ****; ns, no significance).

To interrogate the functional interplay between AAG_score and immune microenvironment composition, we leveraged the CIBERSORT deconvolution algorithm. Analytical outputs revealed significant positive associations (threshold of p< 0.05) between elevated AAG_score values and infiltration levels of activated memory CD4+ T lymphocytes, M0/M1/M2 macrophage polarization states (**Figure 7a**). Conversely, inverse relationships were identified with naive CD4+ T cell populations, regulatory T cells (Tregs), CD8+ cytotoxic T lymphocytes, and memory/plasma B cell compartments. Notably, the AAG_score demonstrated synergistic correlations with both stromal compartment activation indices and immune microenvironment quantification metrics (**Figure 7b**, p< 0.0001). At the same time, the bar chart shows the relationship between gene expression levels and related risk scores in the prediction model, among which XAGE2 presents the strongest correlation (**Figure 7c**). Subsequently, we selected the top seven genes with a closer correlation to the model risk score and further explored their correlation with immune cell enrichment. The results showed that, consistent with previous analysis, the selected genes were closely related to the enrichment of macrophages, regulatory T cells, and dendritic cells (**Figure 7d**, p< 0.05). Furthermore, we evaluated the relationship between ICPs and this prognostic feature. **Figure 7e** illustrates notable differences in the expression of 23 immune checkpoints between the two risk subgroups. The results showed that the high-risk group had a higher level of immune infiltration (p< 0.05).

### 3.7 Association of AAG_score with TMB, MSI, and CSC scores

**Figure 8:**
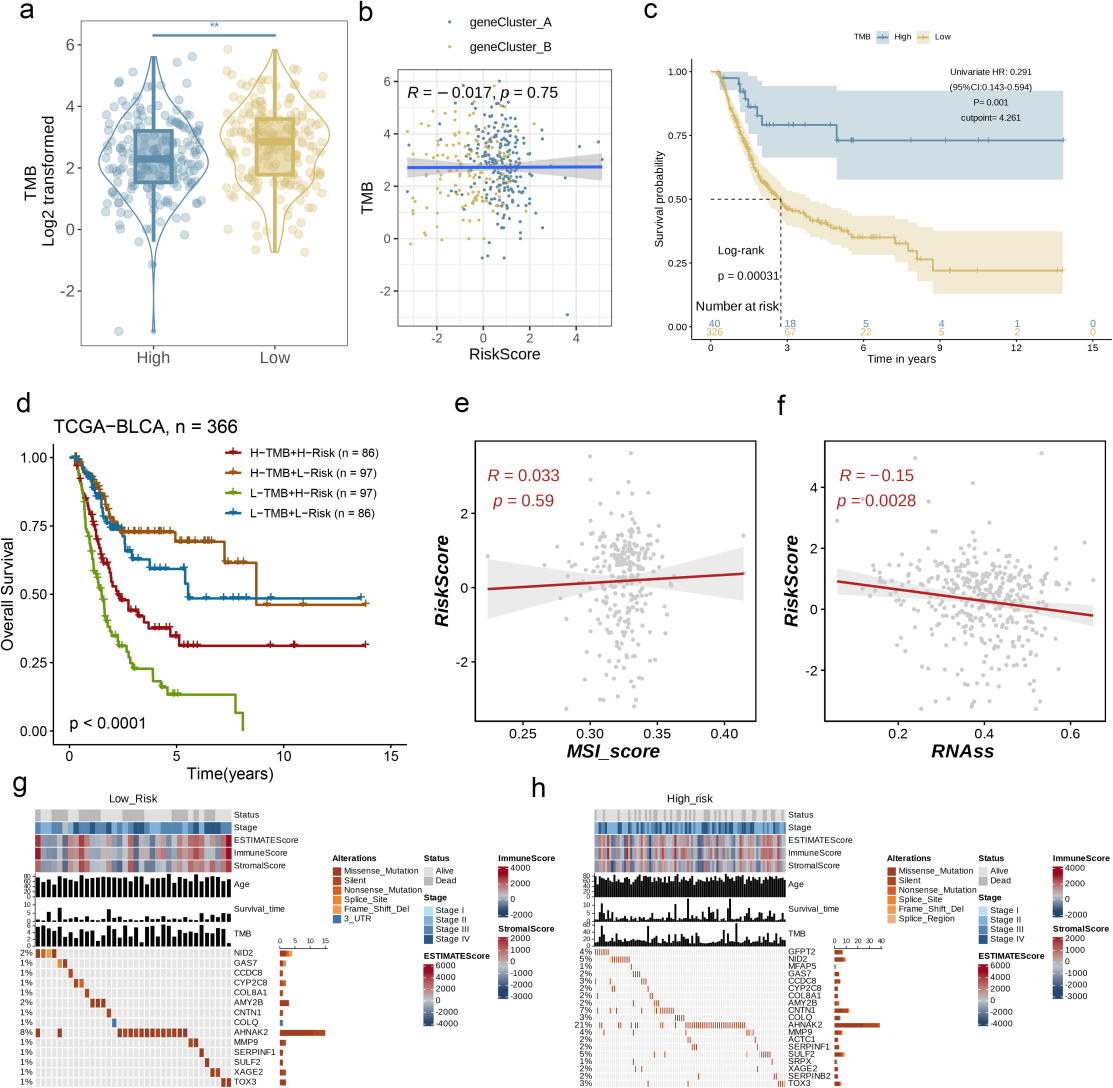
Association of AAG_Score with TMB, MSI, and CSC Scores. **(a, b)** Relationships between AAG_score and TMB. **(c)** Kaplan–Meier analysis of the OS between the low- and high-TMB groups. **(d)** Survival analysis among four patient groups stratified by both TMB and AAG_score. **(e, f)** Relationships between AAG_score and MSIand CSC index. **(g, h)** The waterfall plot of somatic mutation features established with low and high AAG_scores. (p< 0.01 **).

A wealth of studies has established that both TMB and MSI serve as critical predictors for tumor immune responses, with patients exhibiting high TMB or MSI often showing greater responsiveness to ICP inhibitor therapy. Comparative analysis demonstrated marked reductions in tumor mutational burden (TMB) levels within the low-risk cohort relative to high-risk counterparts (**Figure 8a**, p< 0.01), suggesting enhanced immunotherapy efficacy potential in high-risk patients. Subsequent Spearman correlation modeling identified an inverse association between AAG_score and TMB metrics (**Figure 8b**). To delineate TMB-mediated prognostic stratification, we conducted systematic survival evaluation across TMB-defined subgroups, revealing statistically superior clinical outcomes in high-TMB patients compared to low-TMB individuals (**Figure 8c**).

Following this, we performed a survival analysis incorporating both TMB and AAG_score in BC patients, and observed that the prognostic advantage associated with high TMB was neutralized upon the inclusion of AAG_score (**Figure 8d**, p< 0.0001). The correlation assessment results showed that there is no significant correlation between AAG_score and MSI_score (**Figure 8e**, R = 0.033, p=0.59). Cumulatively, these findings indicate enhanced immunotherapy susceptibility in high-risk patient cohorts. We subsequently performed integrated analysis of AAG_score and cancer stem cell (CSC) score to evaluate their correlative interplay in BC biology. As graphically represented in **Figure 8f**, our multidimensional analysis identified an inverse relationship between AAG_score and RNAss, demonstrating that BC specimens with reduced AAG_score values manifest marked stemness characteristics and a poorly differentiated phenotypic state.

Moreover, we examined the differences in the distribution of somatic mutations across various AAG_score patterns within the BLCA dataset. As shown in **Figures 8g, h**, the mutation incidence of AHNAK2 in BC patients was higher than 15% in both risk groups. Remarkably, the high-risk group exhibited a significantly greater likelihood of mutation in these genes when compared to the low-risk group.

### 3.8 Relationships between AAG_score and therapeutic sensitivity

**Figure 9:**
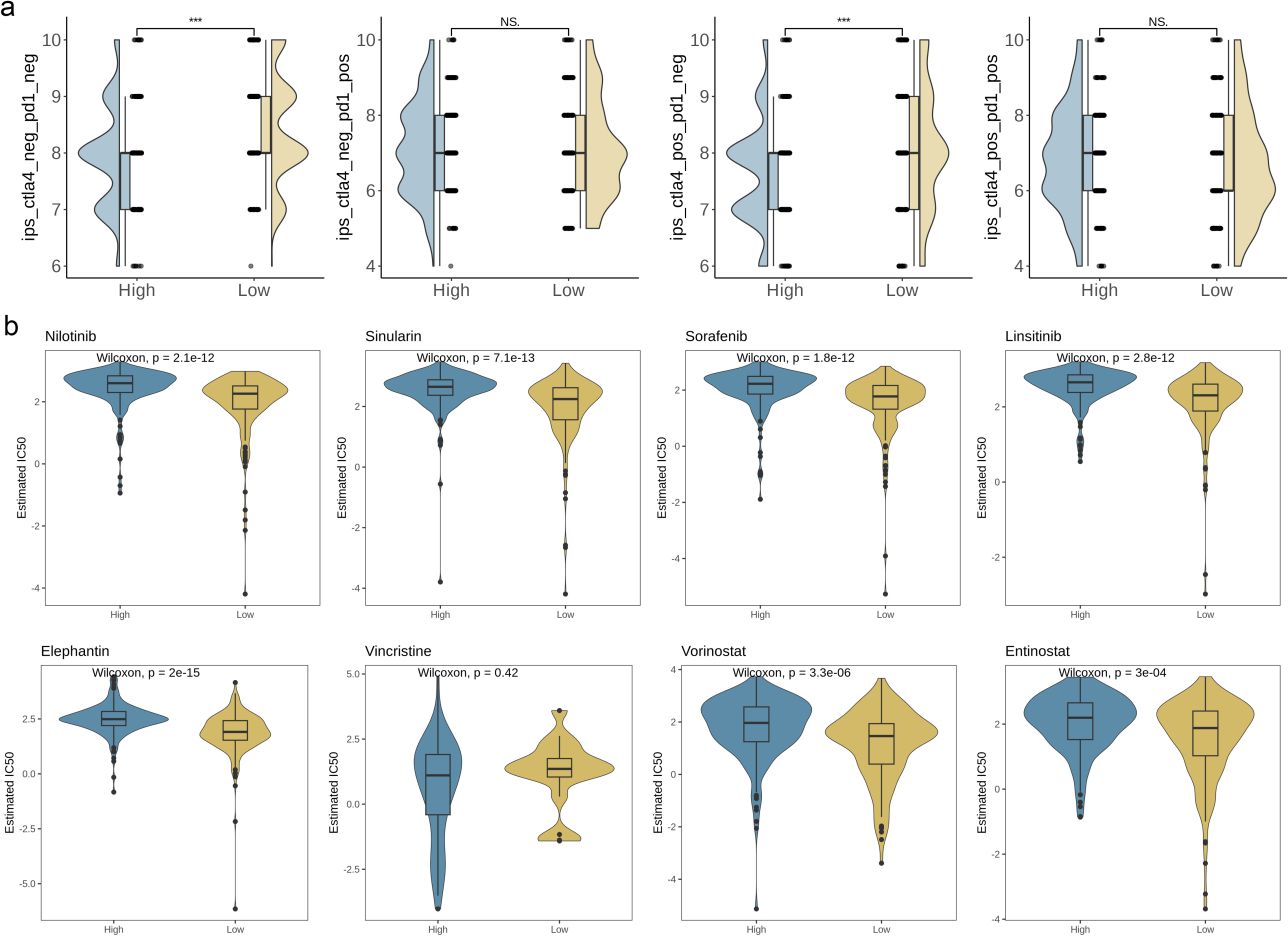
Relationships between AAG_score and therapeutic sensitivity. **(a)** IPS in different AAG_score groups. **(b)** Relationships between AAG_score and chemotherapeutic sensitivity. (p< 0.001 ***; ns, no significance).

In order to assess the immune response in BC patients, we computed the immune phenotype score (IPS) as a predictor of their response to immunotherapy. **Figure 9a** illustrates that the IPS score was notably higher in the high-risk group, implying that these patients may exhibit greater sensitivity to immunotherapy (p< 0.001). To further validate the AAG_score as a potential biomarker for predicting treatment response in BC patients, we assessed the half-maximal inhibitory concentration (IC50) values of commonly prescribed first- and second-line clinical drugs used in bladder cancer patients. The results showed that patients with a low AAG_score showed high sensitivity to multiple chemotherapy drugs, such as Nilotinib, the simulation analog of simvastatin, Sorafenib, Linsitinib, Elephantin, Vorinostat, and Entinostat (**Figure 9b**, p< 0.05). In summary, these research results indicate that the AAG_score is closely related to drug sensitivity and can provide valuable reference for clinicians to choose appropriate chemotherapy drugs for patients.

### 3.9 AAG_score at single-cell levels of BC

**Figure 10:**
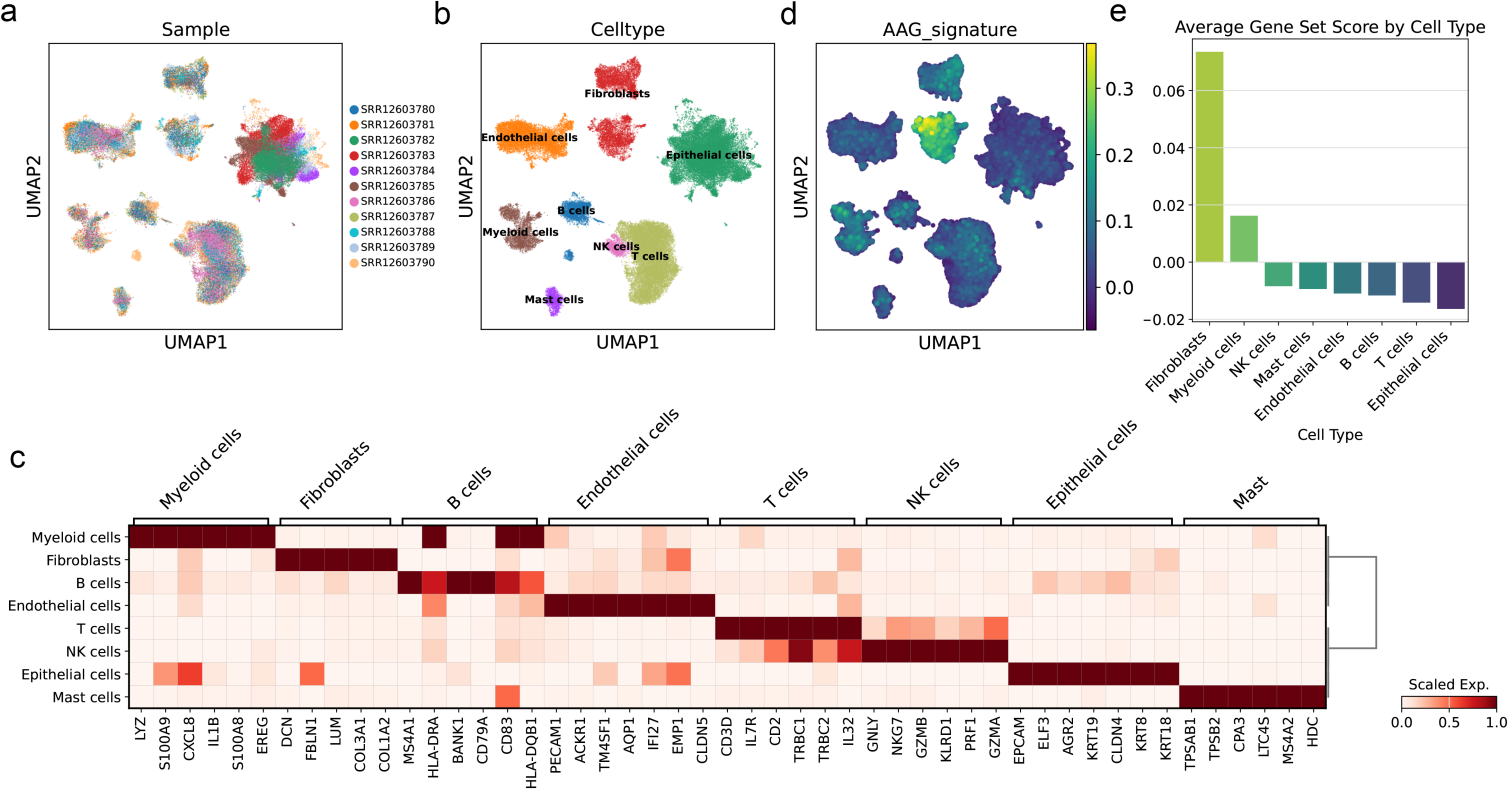
AAG_score at single-cell levels of BC. **(a)** UMAP visualization of single-cells from eleven samples of BLCA. Cells were colored by sample ID. **(b)** UMAP visualization of single-cells from eleven samples of BLCA. Cells were colored by cell type. **(c)** Heatmap of cell marker of each cell type. **(d)** UMAP visualization of AAG_score calculated by scanpy.tl.score_genes function of the Scanpy. **(e)** Bar plot showing the average AAG_scores of each cell type.

We successfully obtained 11 cell subpopulations through UMAP dimensionality reduction (**Figure 10a**). Based on cell type-specific markers reported in previous literature, we conducted cell clustering for all cells, eight types of cells were annotated (**Figures 10b, c**). After that, we applied the prognostic model to the single-cell samples, and the results showed that the AAG_score was specifically elevated in the fibroblast population, while the score was lowest in the epithelial cells, which are more common in bladder tissue samples (**Figures 10d, e**). Among the various cell types present in solid tumors, fibroblasts play a crucial role. They combine with the extracellular matrix of connective tissue and are the basis for maintaining the structural integrity of tissues. Tumor-associated fibroblasts (CAFs) have been demonstrated in prior research to significantly contribute to tumor progression by promoting various processes. The previous research results showed that the high-score group had higher sensitivity to immunotherapy, which means that this prognostic model can well predict the high fibrosis of bladder tissue after the formation of BC, thereby achieving the predictive effect on the progression of BC.

### 3.10 AAG subgroups and traits of 9 sample subtypes in DLBC via clustering

**Figure 11:**
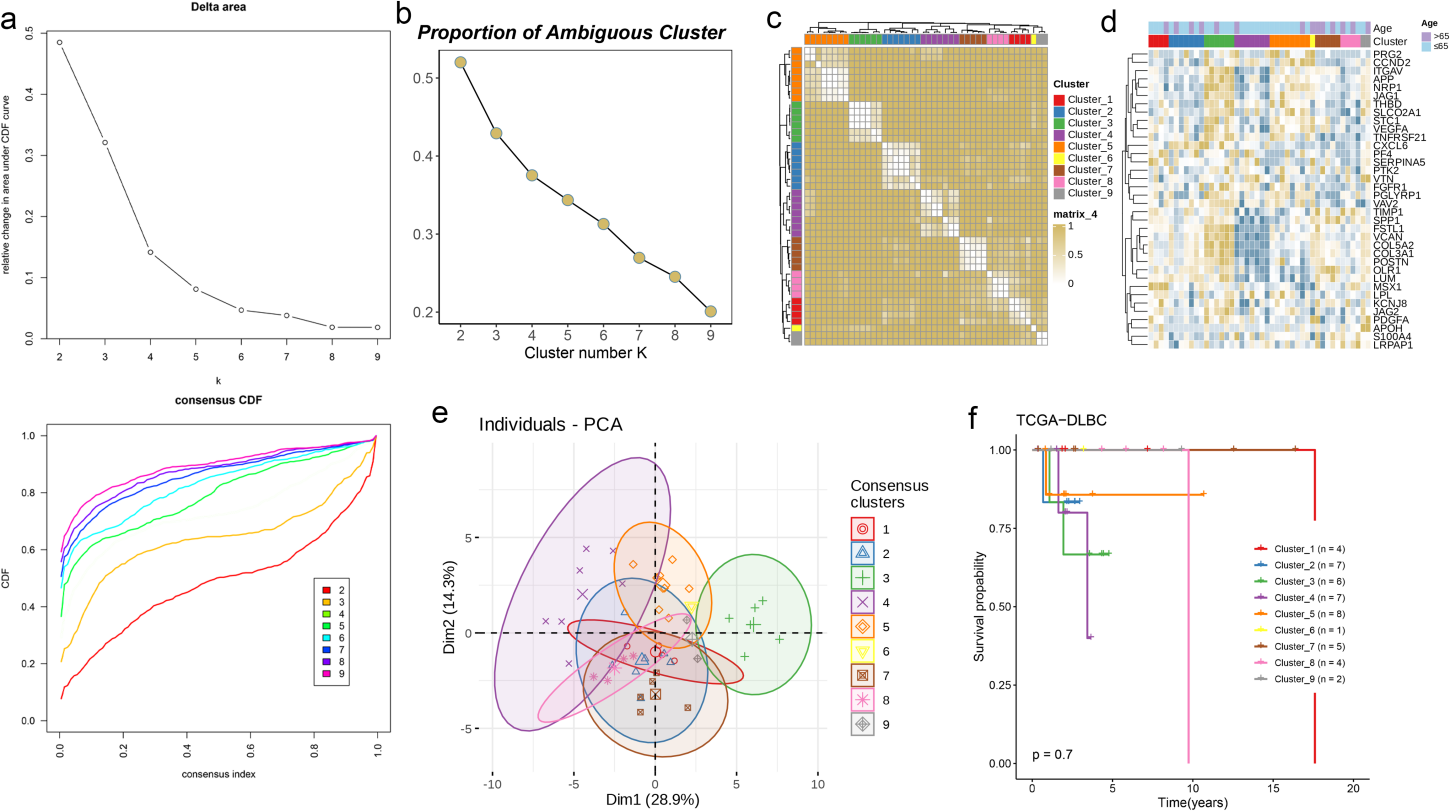
AAG subgroups and traits of 9 sample subtypes in DLBC via clustering. **(a, b)** Consensus CDF curves and PAC analysis indicating the optimal clustering number of 9. **(c)** Consensus matrix heatmap defining nine clusters (k = 9) and their correlation area. **(d)** Differences in clinicopathologic characteristics and expression levels of AAGs between the nine distinct subgroups. **(e)** PCA analysis indicating no obvious difference in transcriptomes between the nine subgroups. **(f)** KM survival analysis of the nine subgroups in DLBC.

Whether solid tumors or non-solid tumors, the rapid proliferation of tumor cells relies on an abundant supply of nutrients and oxygen. Although non-solid tumors (such as leukemia and lymphoma) do not form solid masses, they still depend on angiogenesis to sustain their growth and dissemination. Based on this, we conducted a joint analysis of angiogenesis-related genes and transcriptome data from DLBC. Initially, we performed LASSO regression and multivariate regression analysis on the data (**Figures 11a, b**), followed by PCA to classify the samples into nine clusters. Simultaneously, we incorporated AAG scores and clinical data into each cluster to observe their expression levels (**Figures 11c–e**). Additionally, we analyzed the OS of the nine clusters, but no significant survival differences were observed among them (**Figure 11f**, p = 0.7). This phenomenon may be related to the fact that non-solid tumors typically contain fewer fibroblasts.

### 3.11 NID2 played a carcinogenic role in bladder cancer cells

**Figure 12:**
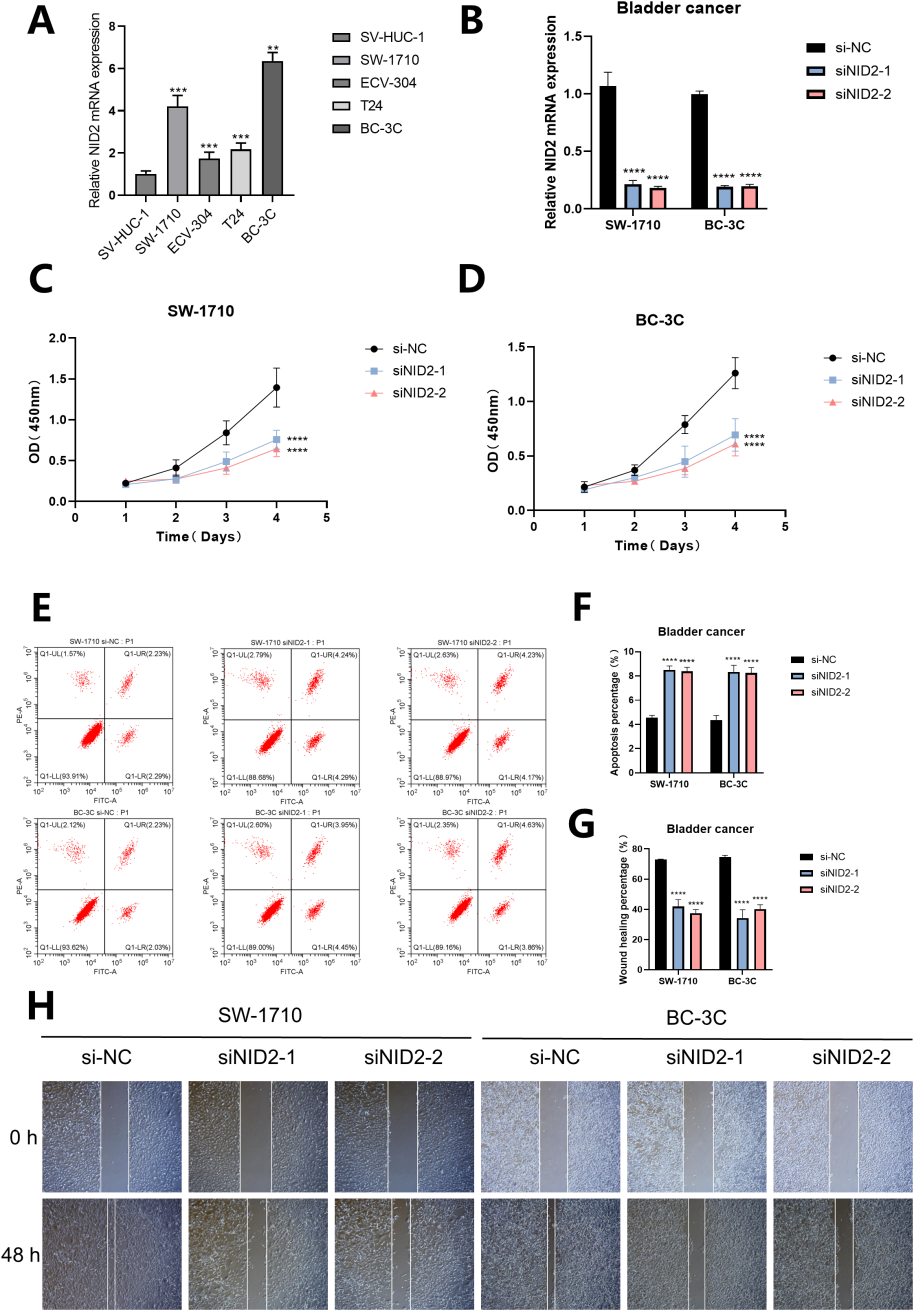
The proliferation, apoptosis and migration of cells after NID2 knockdown in bladder cancer. **(A)** The expression of NID2 mRNA in different cell lines was detected by RT-qPCR. **(B)** The bar chart shows the expression of NID2 mRNA in the SW-1710 and BC-3C cell lines treated with siRNA. **(C, D)** The proliferation of cells was detected by CCK8. **(E, F)** The apoptosis of cells was detected by Flow cytometry. **(G, H)** The migration of cells was detected by Wound healing assay. A *P* value< 0.05 was considered statistically significant (** *P*<0.01; *** *P*<0.001; **** *P*<0.0001; ns, no significance).

For different cell lines, the RT-qPCR results showed that the mRNA expression level of NID2 was at a high level in all four human bladder cancer cell lines, and was significantly upregulated in the SW-1710 and BC-3C cell lines (P< 0.01, **Figure 12A**). Furthermore, by observing the bar chart, it can be found that in the SW-1710 and BC-3C cell lines, the knockdown efficiency and targeting of NID2 are both good (P< 0.0001, **Figure 12B**). The CCK-8 assay results showed that after knockdown of NID2, the proliferation ability of cancer cells was significantly weakened, indicating that NID2 has a promoting effect on the proliferation of bladder cancer cells (P< 0.0001, **Figures 12C, D**). Meanwhile, Flow cytometry detection revealed that when NID2 was knocked down, the percentage of apoptotic cells in the cell line increased significantly (P< 0.0001, **Figures 12E, F**). Finally, the Wound healing assay showed that the migration ability of cells knocked down with NID2 was significantly weaker than that of the NC group (**Figures 12G, H**). Based on the above experimental results, NID2 plays a promoting role in bladder cancer.

## 4 Discussion

Tumor growth and metastasis are highly dependent on neovascularization to supply oxygen and nutrients (32). BC, especially muscle-invasive BC, breaks through the basement membrane by inducing angiogenesis, thereby invading surrounding tissues or metastasizing to distant sites (33, 34). This process is finely regulated by various angiogenic cytokines, such as VEGF, FGF, and PDGF (35–37). Research has demonstrated that the concentration of VEGF in BC tissues is notably elevated compared to normal bladder mucosa, and its expression exhibits a positive association with tumor stage and grade (38). The detection of VEGF in urine can even serve as a non-invasive diagnostic marker, further highlighting the close and inevitable link between angiogenic cytokines and BC (39). Currently, interventions targeting angiogenesis have become a new direction in BC treatment, such as anti-VEGF drugs (e.g., bevacizumab) and tyrosine kinase inhibitors (TKIs) (40–42). However, the clinical efficacy of these drugs, which target only a single pathway or specific immune cells, is extremely limited. Hence, it is of utmost importance to further elucidate the comprehensive impact of AAGs and the infiltration characteristics of the TME under varying combinations of AAGs.

This study involved a thorough examination of the transcriptional changes and expression patterns of AAGs by leveraging data from the BLCA cohort. Our results indicated that while the mutation rate of AAGs is relatively low, there are significant differences in their expression levels between cancerous and adjacent normal tissues, which are closely tied to patient prognosis. Following this, unsupervised clustering techniques were employed to categorize BC patients into two distinct angiogenic subgroups, referred to as Cluster 1 and Cluster 2. Inter-subgroup analyses revealed marked divergence in clinical trajectories, immune infiltration landscapes, and functional pathway activation patterns. Critical to emphasize is the emerging evidence^43,44 that BC-associated genetic mutations exert pivotal regulatory influences on therapy-responsive immunomodulatory mechanisms through underlying molecular cascades.

Leveraging subgroup-defining differentially expressed genes, we computationally stratified two distinct genomic clusters exhibiting divergent clinical profiles, immunomodulatory dynamics, and pathway activation patterns. Through LASSO Cox regression feature selection, we subsequently constructed the AAG_score quantification system to characterize angiogenic subgroup heterogeneity.

Notably, a shorter OS was observed in patients with elevated AAG_scores, implying that a high AAG_score could potentially act as a prognostic marker for poor outcomes. Angiogenesis is a key driver of cancer progression, and its dysregulation is closely associated with tumor aggressiveness, treatment resistance, and adverse outcomes (43, 44). Our GSEA results further supported this notion, demonstrating significant enrichment of pathways related to cancer, proliferation, and metastasis.

AAG_score exhibited a strong association with the clinicopathological features of BC. Upon accounting for confounding variables, our findings demonstrated that the AAG_score independently predicts survival outcomes in BC patients. ROC curve analysis validated its high accuracy in predicting 1-year, 3-year, and 5-year overall survival. Moreover, a high AAG_score often corresponded to a higher clinical stage. Therefore, the AAG_score likely possesses reliable predictive capabilities for patient prognosis. The accumulation of genetic mutations is a key factor in carcinogenesis, which is closely linked to neovascularization. Intergroup genomic profiling identified significant genomic disparity between low- and high-AAG_score cohorts. Consistent with prior evidence^47, elevated TMB levels demonstrated correlative enhancement of clinical prognosis in BC populations. Notably, low-AAG_score specimens exhibited significantly improved clinical trajectories compared to low-TMB counterparts, establishing the AAG_score as an independent prognostic determinant of immunotherapy responsiveness.

The role of immune interactions is pivotal in the tumorigenesis of BC (45). Stromal cells and immune cells, as integral components of the TME, are closely associated with the clinical characteristics and prognosis of BC through related immune scores and stromal scores (46, 47). In this study, we employed a specialized algorithm to compute the immune and stromal scores. Analytical data demonstrated that BC specimens with elevated AAG_score displayed statistically elevated immune/stromal microenvironmental indices compared to low-score counterparts. These collective observations imply an intricate coupling between angiogenic activation and tumor microenvironmental remodeling, mechanistically modulating BC malignant transformation and metastatic dissemination. Notably, the high-AAG_score cohort manifested substantial infiltration of naive B lymphocytes, CD4+ memory T lymphocytes, M0/M1/M2 macrophage subtypes, and neutrophil populations.

Previous research has demonstrated that these cells correlate with poor clinical prognosis, a finding that aligns with the generally worse clinical outcomes seen in the high AAG_score group in our study (48–51). Several studies suggest that angiogenic factors may function as immunomodulators, with the immune system playing a role in carcinogenesis by promoting pathological vascularization (52, 53). Consequently, targeting angiogenesis emerges as a promising regulatory approach for BC immunotherapy.

Currently, the resistance of BC to chemotherapy is increasing (54, 55). Additionally, for immunotherapy to be effective, specific biomarkers are needed as predictive indicators. Currently, TIDE and IPS scores have been developed to assess the therapeutic response to ICIs. Based on this, we found that BC patients with low AAG_scores had lower TIDE scores, indicating that they are more sensitive to anti-PD-1 and anti-CTLA-4 therapies. Meanwhile, patients with low AAG_scores also showed higher sensitivity to most chemotherapy drugs.

BC exhibits high heterogeneity, which is one of the key mechanisms underlying tumor survival and evolution (56, 57). In the tumor microenvironment of BC, the distribution and functional status of immune cells, fibroblasts, and other stromal cells significantly impact BC progression (58, 59). Recent research has emphasized the role of fibroblasts in critical physiological processes, including tumor angiogenesis and the remodeling of the extracellular matrix. Moreover, the extent of fibroblast infiltration in BC tissues is strongly associated with a poor prognosis in patients (60, 61). We analyzed single-cell sequencing data of BC and applied our constructed predictive model to this data. The results showed that AAG_scores were significantly higher in fibroblasts than in other cell populations. This result not only confirms previous studies but also fully demonstrates the accuracy of our predictive model. Meanwhile, to test the model’s generalizability, we applied it to a non-solid tumor—diffuse large B-cell lymphoma. However, the model did not show a close association with prognostic indicators. This may be related to the lower number of fibroblasts in diffuse large B-cell lymphoma. Finally, the wet experiment results confirmed that NID2 promotes the proliferation and migration of bladder cancer cells, inhibits apoptosis, and plays a pro-cancer role.

This study, for the first time, systematically revealed the pro-cancer function of NID2 in bladder cancer models: it showed significant mRNA high expression in bladder cancer cells, and after specific knockdown by siRNA, the cell proliferation ability was significantly inhibited. This provides a theoretical basis for subsequent targeted therapy research.

This study has certain limitations. When exploring the clinical value of the AAG_score, the study included a relatively limited number of clinical variables. Subsequent studies should incorporate more relevant clinical variables to more comprehensively and deeply explore its clinical significance. In this study, functional experiments focused on the assessment of cell proliferation and did not involve studies of other tumor-related phenotypes, such as the aggressiveness or apoptosis of tumor cells. Therefore, while cell proliferation experiments provide important information for evaluating treatment efficacy, they are not fully representative of the overall characteristics of the tumor. Future studies need to consider multifaceted tumor-related phenotypes and comprehensively evaluate the effects and mechanisms of potential therapeutic strategies. Additionally, to better elucidate the connection between the risk score and the TME, and to further corroborate our findings, it is essential to conduct *in vivo* and *in vitro* experiments in conjunction with comprehensive prospective studies.

## 5 Conclusion

To summarize, we carried out a systematic and thorough investigation of AAGs, uncovering a multifaceted regulatory mechanism that profoundly influences the TME, prognosis, and clinical features of BC patients. Simultaneously, we demonstrated the utility of AAGs as biomarkers for predicting treatment response. Our research underscores the substantial clinical relevance of AAGs and lays the groundwork for personalized treatment strategies in BC patients.

## Data Availability

The raw data supporting the conclusions of this article will be made available by the authors, without undue reservation.
